# An exploratory study combining Virtual Reality and Semantic Web for life science research using Graph2VR

**DOI:** 10.1093/database/baaf008

**Published:** 2025-05-20

**Authors:** Alexander J Kellmann, Sander van den Hoek, Max Postema, W T Kars Maassen, Brenda S Hijmans, Marije A van der Geest, K Joeri van der Velde, Esther J van Enckevort, Morris A Swertz

**Affiliations:** Department of Genetics, Genomics Coordination Center, University Medical Center Groningen, Antonius Deusinglaan 1, Groningen, Groningen 9713 AV, The Netherlands; Department of Genetics, Genomics Coordination Center, University Medical Center Groningen, Antonius Deusinglaan 1, Groningen, Groningen 9713 AV, The Netherlands; Department of Genetics, Genomics Coordination Center, University Medical Center Groningen, Antonius Deusinglaan 1, Groningen, Groningen 9713 AV, The Netherlands; Department of Genetics, Genomics Coordination Center, University Medical Center Groningen, Antonius Deusinglaan 1, Groningen, Groningen 9713 AV, The Netherlands; Department of Genetics, Genomics Coordination Center, University Medical Center Groningen, Antonius Deusinglaan 1, Groningen, Groningen 9713 AV, The Netherlands; Department of Genetics, Genomics Coordination Center, University Medical Center Groningen, Antonius Deusinglaan 1, Groningen, Groningen 9713 AV, The Netherlands; Department of Genetics, Genomics Coordination Center, University Medical Center Groningen, Antonius Deusinglaan 1, Groningen, Groningen 9713 AV, The Netherlands

## Abstract

We previously described Graph2VR, a prototype that enables researchers to use virtual reality (VR) to explore and navigate through Linked Data graphs using SPARQL queries (see https://doi.org/10.1093/database/baae008). Here we evaluate the use of Graph2VR in three realistic life science use cases. The first use case visualizes metadata from large-scale multi-center cohort studies across Europe and Canada via the EUCAN Connect catalogue. The second use case involves a set of genomic data from synthetic rare disease patients, which was processed through the Variant Interpretation Pipeline and then converted into Resource Description Format for visualization. The third use case involves enriching a graph with additional information, in this case, the Dutch Anatomical Therapeutic Chemical code Ontology with the DrugID from Drugbank. These examples collectively showcase Graph2VR’s potential for data exploration and enrichment, as well as some of its limitations. We conclude that the endless three-dimensional space provided by VR indeed shows much potential for the navigation of very large knowledge graphs, and we provide recommendations for data preparation and VR tooling moving forward.

**Database URL**: https://doi.org/10.1093/database/baaf008

## Introduction

Data in life sciences and health is increasing in volume and complexity. It is stored in countless places and offered in equally many shapes and forms Often, Semantic Web technology is now chosen to represent these fragmented and heterogeneous data as Semantic Web statements, e.g. using Resource Description Format (RDF) triples, in a “knowledge graph” that can be cross-linked across the web for joined querying, analysis, and visualization. However, querying datasets in VR remains an unexplored challenge. For instance, while a PubMed search for “linked data” or “semantic web” or “RDF” yields over 5000 hits, and a search for “virtual reality” yields over 20 000 hits, a combined search of those terms only yields six hits. This shows that virtual reality (VR) techniques are being discussed in research and health care in the life sciences domain, but that the combination of Linked Data and VR is nearly nonexistent. Furthermore, of the six hits that were found, none of them actually describe the integration of these two techniques.

In a previous work, we developed the Graph2VR prototype to visualize Linked Data triples in VR, offering an immersive experience for users to intuitively explore and interact with Semantic Web data [1]. In this manuscript, we evaluate how Graph2VR performs when applied to realistic life science use cases.

Graph2VR is able to visualize SPARQL “CONSTRUCT” query results in VR, offering an immersive experience for users to explore and interact with data in a virtual environment. SPARQL (SPARQL Protocol and RDF Query Language) is a query language used to retrieve and manipulate data stored in RDF format in the form of triples. SPARQL offers different forms of queries like “SELECT,” “DESCRIBE,” “ASK” and “CONSTRUCT” queries [2]. While SELECT queries return table-like result sets, CONSTRUCT queries return results in a graph structure. In Graph2VR, the user can see these graphs as nodes connected by edges that represent the predicates connecting the nodes. The user can then grab and move the nodes around in a three-dimensional space. The nodes and edges can also be set as parameters in a query to create a template for the SPARQL query, providing a visual and easy-to-understand representation of the query. Graph2VR also allows navigation through complex knowledge graphs by expanding or collapsing edges that connect the different nodes, allowing users to drill down into the data and explore the relationships between different nodes. This is not restricted to one database; it is possible to switch between databases and graphs on the fly and to expand the data with triples from different sources.

Our aim in this study is to demonstrate the use of Graph2VR with realistic scientific data to show its practical application and versatility. To do so, we focus on three distinct use cases selected to evaluate the use of Graph2VR on different types of data in the life science field. The first use case involves the MOLGENIS catalogue, part of the EU-CAN Connect project [3], which visualizes metadata for large-scale cohort studies and real-world evidence data [4]. This catalogue is used by researchers to make their datasets insightful and facilitates cooperation between different studies. The second use case examines the Variant Interpretation Pipeline (VIP), a tool developed by the University Medical Center Groningen Genomics Coordination Center for prioritizing and interpreting potentially disease-causing genetic variations from next-generation sequencing results in diagnostics [5]. The third use case explores data from Drugbank [6] and PubChem [7], focusing on the intersection of Dutch drug names with Anatomical Therapeutic Chemical (ATC) codes. We believe that Use case 1 represents databases involving complex conceptual knowledge with many different elements, whereas Use case 2 represents a highly detailed molecular data use case and Use case 3 represents studies that involve huge data graphs spanning multiple databases.

Below we first describe the methods and materials used for all use cases. Subsequently, we describe the experimentation with each use case. For each, we first developed different scenarios to evaluate the added value of Graph2VR. We then selected and converted data relevant to the use case into RDF format. Finally, we uploaded the RDF into a Virtuoso server, visualized the different datasets in Graph2VR, and evaluated how this served the use cases. For each use case, we provide specific observations, followed by a cross-use case discussion summarizing our findings and recommendations.

## Materials and Methods

To run Graph2VR, we used a Quest 2 headset (the Quest 3 also works), a Windows PC with a modern graphics card (GTX 1060 or higher), and Docker to run a Virtuoso server. The full description of the requirements can be found on GitHub [8]. We also expect users to have some knowledge of the Semantic Web and SPARQL. For each of the three use cases, we prepared the data, uploaded it to SPARQL endpoints, configured Graph2VR, and then explored the results. We used the dockerized versions of the Open Link Virtuoso server [9] to upload the data for each use case into separate SPARQL endpoints. To use the three datasets within Graph2VR, the existing data had to be translated into a graph database format like RDF or Web Ontology Language. This process varied across the different projects. After preparing each dataset, we uploaded the dataset to a Virtuoso server, enabling access to the data with Graph2VR via the SPARQL endpoint. The server and starting query that Graph2VR uses could then be configured. After selecting a starting point in the graph, the user could use the menu to expand the graph and the controls to interact with the graph and move through space. Since the setup of the Virtuoso server and configuring Graph2VR are consistent across all use cases, below we first explain these processes in general terms, followed by a detailed description of each use case.

### Setting up a local Virtuoso Server using Docker

The choice of Virtuoso as the SPARQL Endpoint for Graph2VR was driven by its ability to: (I) efficiently host large amounts of triples and (ii) serve as a backend for Graph2VR. A Virtuoso server can be used to load and expose Linked Data via a SPARQL Endpoint (we did not explicitly implement support for other servers, although DotNetRDF would offer the functions to connect to them). More information can be found in the GitHub Readme in the Graph2VR

Manual [8, 10] A Virtuoso server as a backend can be set up via Docker. Virtuoso’s web interface can also be used to upload small .owl or .rdf files. Larger files must be loaded via the Virtuoso bulk loader.

### Setting up Graph2VR

Graph2VR can be downloaded from GitHub via https://github.com/molgenis/Graph2VR. There are currently two versions: a stand-alone version in the form of an .apk file that can be loaded on the Quest 2 headset and a Windows version in the Graph2VR.zip file that can be unzipped and run on Windows [1, 8]. While the stand-alone version does not require that the headset is connected to a computer, the Windows version requires a continuous connection between the computer and headset via cable or WiFi. The advantage of this is that the computer’s graphics card can be used, allowing for better performance and the ability to handle more nodes simultaneously. Use of the stand-alone version requires uploading the .apk file or Graph2VR save files to the VR headset (see Figure A.11). Sidequest, a third-party program, allows access to the Quest 2 headset to upload the .apk file [11].

The settings.txt file can be used to configure Graph2VR. It is used to set the SPARQL Endpoint, to optionally limit the queries to a graph, and to define a query for the initial graph. It can also be used to specify additional databases to switch to. Switching between graphs can be done via the settings menu. For more details, see the Graph2VR manual [10]. For the stand-alone version, the settings file needs to be stored in the folder (”sdcard/Android/data/com.Graph2VR.Graph2VR/files”) on the headset. Graph2VR creates this folder when a save state is created. If the uploaded settings file is not a valid settings file (e.g. not a valid JSON file), Graph2VR will apply internal default settings. The consequence of this is that the initial SPARQL query needs to be JSON-encoded. Only CONSTRUCT queries can be used as initial queries. While certain functionalities (such as BIND, MINUS, or subqueries) are not yet implemented as options within Graph2VR’s GUI, these can still be used within the initial query. An explanation of most settings of the settings.txt can be found in section E.4.

### Preparing the use cases

We prepared the three use cases described in the “Introduction” section following the same procedure. First, we held a workshop with experts associated with the use case in which we defined what data might be useful and what potential objectives of the Graph2VR application would be relevant. This resulted in a list of queries in the form of user scenarios similar to “as a x I would like to y in order to z” in which “x” represents a specific user persona, “y” describes an activity, and “z” defines the envisioned impact. Subsequently, we prepared the RDF dataset and uploaded that into Virtuoso. The datasets were structured and semantically annotated to suit each user scenario. The details for each use case are described below. We then entered the main part of the Graph2VR evaluation by having a second workshop in VR. One of the participants operated the headset, while the others could see VR headset’s view on a computer monitor. For the first use case, we observed errors in the rdf export of the dataset that was addressed by fixing the data export in Molgenis. Each use case experiment was completed by discussion of the findings. In the “Results” section, we summarize the findings for each use case.

### Experimental Setup

The lead authors reached out to experts within associated projects and invited them to participate in the project and become co-authors. The background of these users was a combination of bioinformatics, i.e. some technical background, and domain knowledge, e.g. being an expert in genome data analysis. These experts had seen demos of Graph2VR or even participated as test users in the Graph2VR usability study [1]. During the experiment, in most cases the “user” was actually a combination of a developer, who operated the Graph2VR controls, and a domain expert, working together to achieve the use case result. In the manuscript, we refer to either or both of these roles as the “user” for readability. We conducted brainstorming sessions to identify use cases, described the necessary steps, built the required data, and converted it into RDF format. We then performed a test run (half a day) with a group of 3 to 4 people to execute the test scenario. This process typically revealed issues like missing data or relationships, which led to three or four iterations until a complete use case could be demonstrated. We included screenshots in the manuscript to illustrate the user experience. We evaluated the performance of VR compared to 2D visualization subjectively by asking the test users. Note that we did not prioritize the readability of the text because in VR, everything looks larger and cropping the image would make it impossible to see the bigger picture and the spacious aspect of VR.

## Results

Below we report the results from the use case studies. For each use case, we provide the background of the case and a description of the usage scenarios. We then describe the data preparation and conversion into RDF. Subsequently, we describe the main part of the use case studies: the application of Graph2VR to these data. Finally, we summarize observations made during the use case.

### Use case 1: Finding interoperable cohort data

#### Use case 1: Background

Use case 1 is driven by the desire to integrate data from multiple populations for joint analysis. In the past decades, large cohort studies have collected health-related data from large numbers of individuals to study complex effects on health and disease. To increase the sample size and diversity of the data, and therefore the statistical power, researchers want to share, combine, and harmonize data from different studies and geographic locations. Therefore, many projects are now cataloguing these cohorts [4] and describing the harmonization of their heterogeneous variables onto agreed-upon standard variables as the basis for integrated analysis of data from multiple cohorts [12]. These catalogues contain only metadata (not the privacy-sensitive data itself), but they can guide researchers on how to find and request data access to the sources they require for pooled data analysis.

#### Use case 1: Scenarios

Based on discussions with the domain experts, we selected two main scenarios for Graph2VR. The first scenario is focused on finding and grouping resources (cohorts, databanks, etc.) to match a research topic, based on the idea of a principal investigator (PI) who wants to create a research project/consortium. In this scenario, the user needs to first navigate the topics available and select a topic (or topics) of interest, e.g. “smoking” or “blood pressure” or “tobacco.” From this topic, the user then needs to navigate to the resources linking to these topics (using the keywords). In this scenario, the resource would be a cohort that the PI might want to contact in the end. Based on this result, the user will want to make a query to find all the other resources that have the same matches. Subsequently, the user could annotate the resources into a new graph.

The second scenario goes one step further and is focused on identifying data variables available in each of the cohorts as a basis for the study execution. Here, the user again selects a topic of interest, but instead of browsing for cohorts, they navigate to the variables defined on this topic. This would allow the PI to assess to what extent data are readily available, either because it is collected directly in the relevant form or because data harmonization has already been done.

#### Use case 1: methods and materials

##### Data

As a dataset, we used a sample from the catalogue that the MOLGENIS group works on in EU projects such as EUCAN Connect, LifeCycle, European Human Exposome Network, and IMI Conception [13–16]. The catalogue is stored on a server that runs on the EMX2 version of MOLGENIS https://molgeniscatalogue. org. EMX2 allows automatic export of data in the form of RDF, and several of the metadata elements were already encoded using the underlying URIs. Therefore, the export of the catalogue data was done just using the export functionality of Molgenis. To ease user understanding, we used only a small random subsample of the data, containing only a few cohorts.

##### RDF representation

The first automatic RDF export of the catalogue data proved to be rudimentary, with the structure of the RDF in the catalogue insufficient or malformed. For example, the same URI was used simultaneously as a class, individual, and predicate, something that should not happen unintentionally. As this led to several issues, we worked with the EMX2 team to improve the RDF export and finally imported the RDF file into a Virtuoso server. We then defined a starting query in the settings of Graph2VR. While the starting query had to be set via the Unity editor in the settings singleton in earlier versions of Graph2VR, it is now possible to edit the settings via the file settings.txt (It is necessary to encode the SPARQL query as JSON. We used an online tool to encode the SPARQL query [17]) without using Unity.

#### Use case 1: Results

##### Scenario 1: Finding cohorts containing “smoking” data

In the Molgenis Catalogue use case, we decided to start with a class hierarchy with a depth of 1, similar to the class hierarchy in Prote´ge´ [18]. To do so, our starting query for Graph2VR was written as follows:

PREFIX rdf: <http://www.w3.org/1999/02/22-rdf-syntax-ns#> PREFIX owl: <http://www.w3.org/2002/07/owl#>
PREFIX rdfs: <http://www.w3.org/2000/01/rdf-schema#>
Construct{
   ?superclass rdfs:subClassOf owl:Thing.
   ?superclass rdfs:label ?label.}
WHERE {
   ?superclass rdfs:subClassOf owl:Thing.
   Optional {
     ?superclass rdfs:label ?label.
  }
}

**Figure 1.**
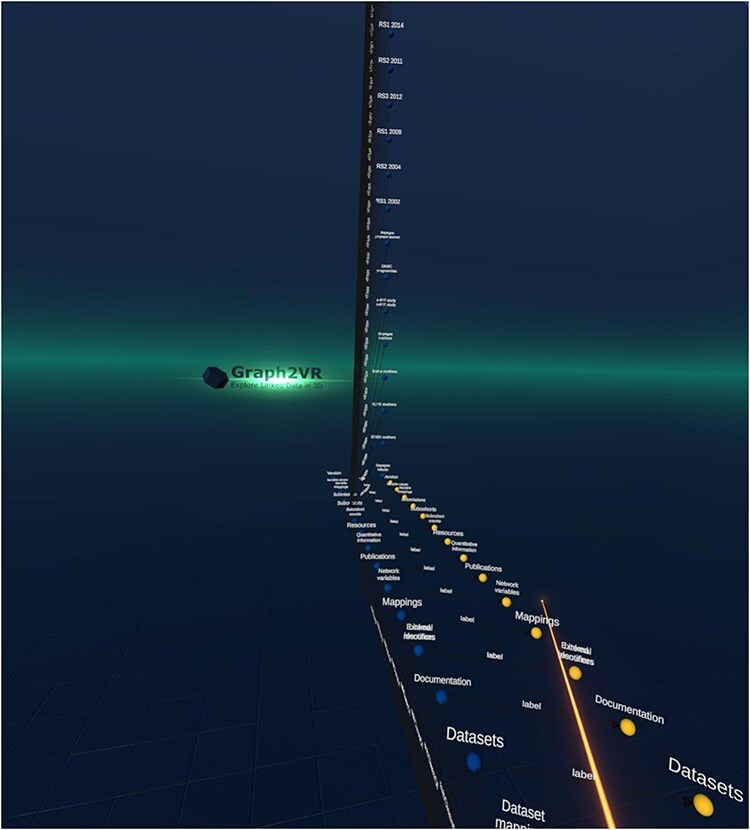
Screenshot of the Molgenis catalogue’s class hierarchy in Graph2VR. The instances of the “subcohorts” are expanded.

The result was a hierarchy showing all the data classes available in the catalogue (see Fig. 1).

**Figure 2.**
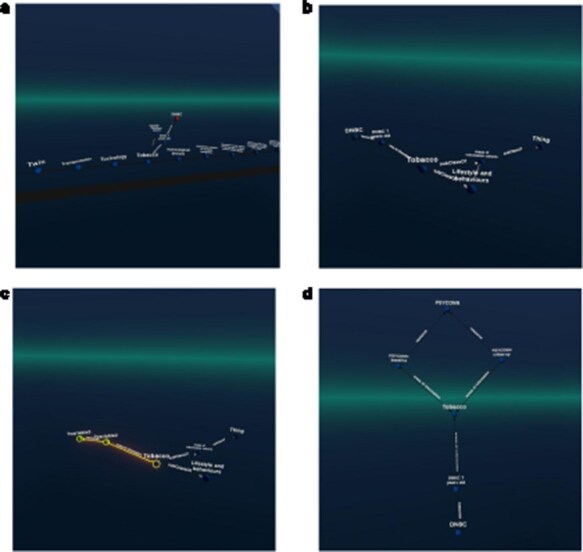
An example figure showing the selection of the topic “tobacco,” followed by navigation to the “DNBC” cohort. The users then specified a query pattern and saw that the PSYCONN cohort was also linked to the same topic in this example set (the original catalogue holds many more records). (The labels were requested manually in Graph2VR).

The user was then able to locate “the” Areas of information cohorts’ class and therein navigate to the topic “Tobacco.” (Within the catalogue, all information related to smoking is tagged with tobacco.) From this topic, the user could navigate to one of the cohorts containing data on this topic, in this particular example, the DBNC study. Based on this example, the user could then create a query pattern that limits the topic to “Tobacco” and then find all cohorts with the same pattern (see Fig. 2) to display them instead of the raw URIs.

##### Scenario 2: Finding datasets with variables harmonized with “smoking”

**Figure 3.**
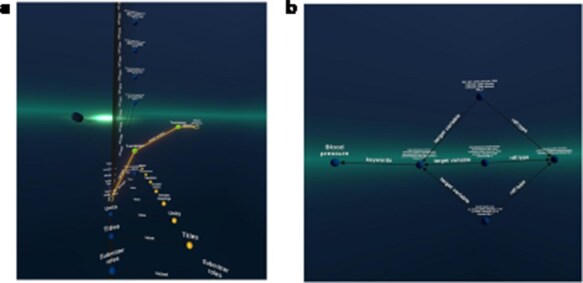
How the user made a query pattern for the variable “blood pressure” and then found all variable-mappings that fulfil this condition. There are three cohorts (G21, ENVIRONAGE, and GENR) in this sample dataset that match, as seen in the middle diamond. This example data was taken from the LifeCycle project. For a complete study overview, see [14].

In the second scenario, the user started with the “Variable mappings” and aimed to find data harmonizations that had the harmonized variable of “Blood pressure” as a target. Here, the user navigated from “Variable mappings” to the “target variable” to those target variables that have “Blood pressure” as their main keyword. Subsequently, the user navigated to the “source variables” of that harmonization and then on to the datasets that hold those variables (see Fig. 3). Based on this example graph, the user can define a query pattern in order to retrieve all other source variables with similar mappings, which is a way to identify all the “smoking”-relevant datasets in the catalogue. Figure 3 shows how such a query pattern is defined.

#### Use case 1: Evaluation

In the catalogue RDF representation, the user was sometimes misled by the domain or range predicate information. In SPARQL, the predicate rdfs:domain should define the class of an individual used as a subject when using a specific predicate. Similarly, rdfs:range is used to limit the permissible input values for the object for forming valid triples with the respective predicate. While Graph2VR is agnostic about the meaning of any predicates for expanding the graph, users might come across incoming predicates like rdfs:domain or rdfs:range. These lead to nodes describing predicates instead of classes, individuals, or literals.

In addition, we observed that metadata collected at the level of variables is relatively sparse. While cohorts generally have huge data dictionaries, these are not annotated against a common ontology. It is only in cases of data harmonization that a lot of effort is spent on mapping the harmonized variables to ontologies. Therefore, the second scenario in this use case is rather artificial. A more realistic use case would be for new researchers to search for previously harmonized variables.

### Use case 2: DNA variant interpretation pipeline

#### Use case 2: Background

Genome diagnostics is the process of analyzing the DNA of patients with a suspected heritable disease. The patient’s DNA is sequenced, and current methods can now measure all genes (i.e. protein-coding DNA, also called whole-exome sequencing) or even all DNA (whole-genome sequencing). The DNA measurement is then compared against the known human reference genome to identify differences, which typically yields 1000 to 100 000 DNA differences called variants. Finding the correct diagnosis is a process of matching diseases, genes, phenotypes, and variants in genes, which involves connecting different data sources. We speculated that visualizing relationships between these different layers of information in VR could speed up the process of prioritizing disease-causing variants. As a basis for this use case, we used the MOLGENIS VIP. VIP uses a decision tree that applies different methods based on best practices from genome diagnostics and research. VIP starts by checking for confirmed variants from databases, then filters for only “rare” variants (those not commonly observed in the general population), and finally continues with statistical methods. If that does not work, VIP uses machine learning methods like CAPICE to predict how a genetic variant should be classified [19]. By applying the decision tree, VIP is able to: present a shortlist of variants that might be relevant to rare diseases; annotate each of these variants with a suggested classification of either Pathogenic (P), Likely Pathogenic (LP), Variant of Unknown Significance (VUS), Likely Benign (LB) or Benign (B); and apply filters to keep only P/LP/VUS variants for expert evaluation [5]. The suggested list of potential disease-causing pathogenic variants is then interpreted by lab specialists who make a final genetic diagnosis. A clinical geneticist then communicates the diagnosis to the patient. Knowing the genetic diagnosis may help to provide a prognosis and even to select an appropriate treatment.

#### Use case 2: Scenarios

Together with the use case experts, we chose three scenarios to use Graph2VR for joint analysis of a cohort of multiple patients with an unclear genetic diagnosis. We speculated that analysis of such unsolved “cohorts” would result in the most interesting questions where visualization as graph in VR could provide unexpected insights [20]

- The first scenario is to find and explore affected genes (i.e. genes with causal variants) that are shared between patients. Here, the Graph2VR user aimed to shortlist relevant DNA variations that are potentially associated with a rare disease, find the associated genes, and view them in groups.
- The second scenario explored patients by clustering them based on their phenotypes. In this scenario, given a group of patients, the Graph2VR user had to find out what phenotypes have been observed for each patient and overlap them. The overall goal was to identify which phenotypes are shared among many patients (disease-specific), which occur rarely, and which occur specifically in disease-affected individuals.
- The third scenario aimed to understand if a patient cohort could be clustered based on the differences in phenotypes in relationships with their causal DNA variants. Here, given the group of patients, the Graph2VR user had to find out what phenotypes are associated to the observed genes and cluster these. The overall goal of this scenario was to identify new genes that are not yet well documented to be linked to disease, which could later be used to short-list DNA variants for individual patients.

#### Use case 2: methods and materials

##### Dataset

Because patient data is sensitive, we used a public dataset for this evaluation. We extracted a subset of patients from the Trujillano *et al*. whole-exome sequencing of 1000 cases (see “Supplementary Table S3” of [21]), specifically patients with the phenotypes “Visual impairment” (HP:0000505) or “Cerebral visual impairment” (HP:0100704). We then synthesized realistic data for the use case by combining these patient data with variants from the general population cohort GoNL [22]. For this, each patient was run through the Ensembl Variant Effect Predictor, after which they were enriched with variants from GoNL, before being processed by VIP [23]. For reproducibility, we have uploaded our scripts on Zenodo with a README file detailing the exact process [24].

##### RDF representation

**Figure 4.**
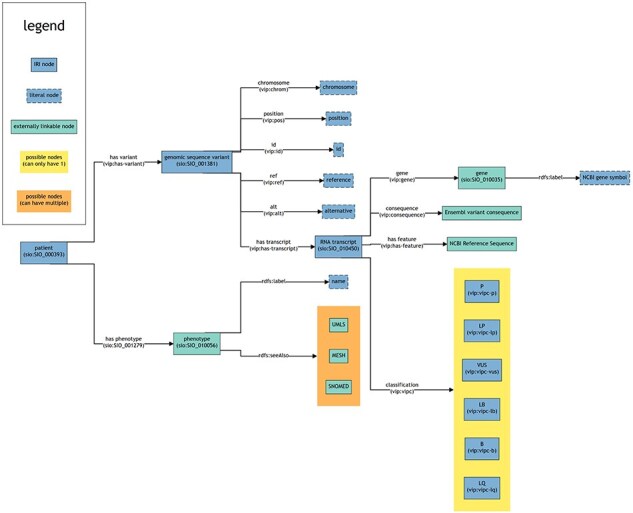
This figure provides a schematic overview of the structure and interconnections within the RDF data (turtle files) generated from VIP VCF files including potential references to external sources. Each box represents a node. Each line represents a predicate. The arrow points towards the object of a triple (subject—predicate—object). The text within the brackets of a box shows the rdf:type of that node. For the lines, the text within brackets represents the IRI used as predicate to link nodes. Note that multiple objects are allowed for certain subjects, even though this is not explicitly mentioned in the figure (e.g. a patient can have multiple variants).

Part of the output from VIP was converted to RDF Turtle files using a custom application [25], including features relevant to the use case as described below. This custom application takes a VIP output .vcf file and a VIP sample-sheet file containing patient metadata, together with two additional files to enrich this data, and then converts them into a Turtle RDF file. These two enrichment files are an Ensembl glossary .owl file and a Human Phenotype Ontology (HPO) .owl file. These are used to add relevant information and/or convert non-RDF data to RDF (IRIs). The output Turtle files were then added to the Virtuoso server [24, 26–28]. These are used to generate relevant Internationalized Resource Identifiers (IRI) for Semantic Web usage and to retrieve additional information. Where possible, pre-existing standards and IRI were used to define the information. An overview of the namespaces used and what they were used for can be found in Supplementary Appendix Table C.1. The design of the final RDF output from a VIP .vcf file is shown in Fig. 4. The VIP vcf files do contain additional fields (such as more data about individual transcripts). These were not included, as they were not relevant for this use case. The generated RDF data was then loaded into both GraphDB and Virtuoso (for Graph2VR) [29]. Within GraphDB, several SPARQL queries were written so that a visual comparison could be made to investigate how a 3D virtual environment could provide added value compared to 2D visualizations on a screen.

#### Use case 2: Results

##### Scenario 1: Cluster patients per gene

**Figure 5.**
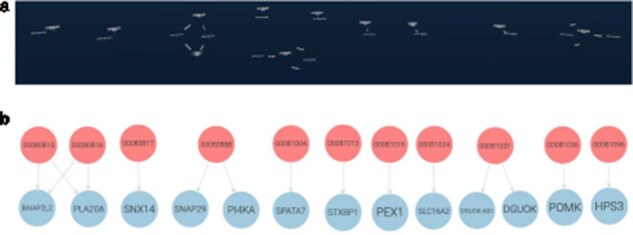
(a) Graph2VR using automatic layouts (first Class hierarchy layout, then 3D Force directed). Even though the graphs in the screenshot look very small, they are only spread out, and the user can easily navigate closer in VR to interact with them. (b) GraphDB manual layout where the red nodes represent patients and the blue nodes genes. Individuals in the dataset, clustered per gene (the order of the graphs in Graph2VR and GraphDB varies).

For the VIP data, we developed queries that can identify individuals who share variations in the same gene. We visualized the results in both Graph2VR and GraphDB (see Fig. 5). Individuals sharing variations in the same gene are likely to experience similar health issues (or benefits) due to these variations, particularly if they have the same variation. This is because such variations can directly alter the function or structure of the protein(s) the gene encodes, which can have various implications. Figure 5 illustrates that the same relations can be displayed in Graph2VR and GraphDB. In Graph2VR, the automatic layouts were used to position the nodes, while in GraphDB, they were placed manually. The advantage of the GraphDB representation in this visualization is that nodes are colored differently based on their rdf:type, so it is immediately visible which node belongs to which group [30]. This has certain limitations, however, for example if a node has multiple rdf:type’s, or with federated querying, where it might not be possible to automatically process the necessary information. A distinct random coloring that is consistently used for each rdf:type of a node is something also used by other tools, such as Gruff [31]. Currently, the coloring of nodes and edges in Graph2VR is mainly based on the VOWL schema. As a result, however, both individual people and genes are treated as instances in this visualization, and both are represented as blue nodes with labels. The labels of the nodes are written within the nodes in GraphDB and below the nodes in Graph2VR.

##### Scenario 2: cluster patients per phenotype (with more than two patients per phenotype)

**Figure 6.**
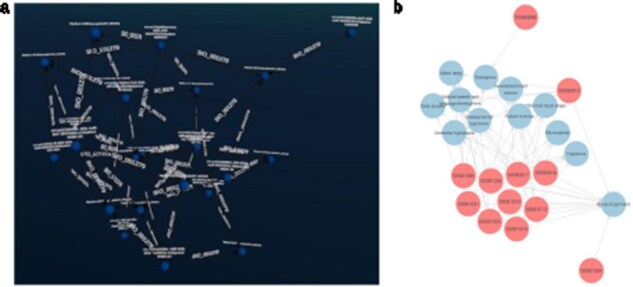
(a) Graph2VR layout. (b): GraphDB default layout. Red nodes represent patients. Blue nodes represent phenotypes. Clustering of individuals with the same phenotype (minimum of two per HPO) in Graph2VR 3D force-directed layout (a) and in GraphDB automatic layout (b).

Patients can manifest multiple disease phenotypes, which they can share with other individuals. This can be visualized in a network of patients and phenotypes, where shared phenotypes lead to a denser network (see Fig. 6). Using this visualization, it is evident that patients do not necessarily share the same cause of their phenotypes. A cluster of individuals may share many of the same phenotypes, hinting that they all have the same or related diseases, while other patients only share one phenotype and most likely have a very different disease. The subset was filtered on “Visual impairment” (HP:0000505) and “Cerebral visual impairment” (HP:0100704), explaining the high number of patients associated with “Visual impairment.”

##### Scenario 3: closing the loop with DisGeNET

**Figure 7.**
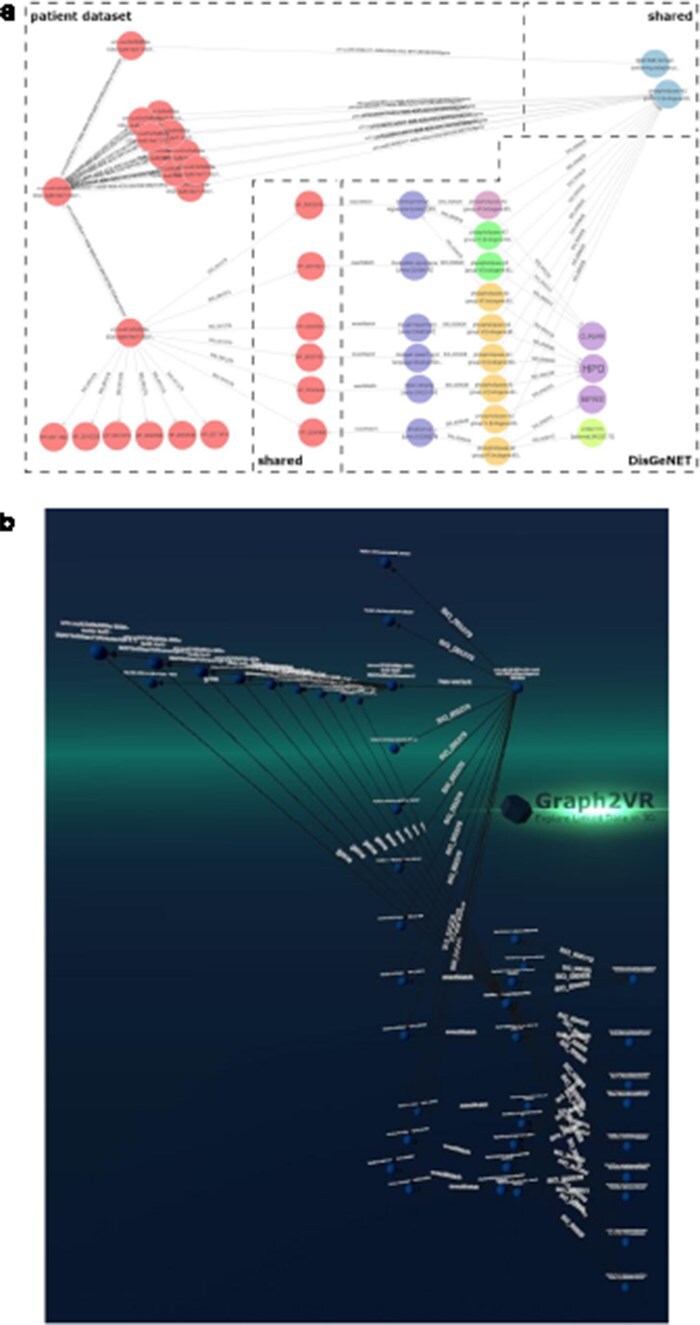
(a) GraphDB layout after manually ordering the nodes. An overlay was added to distinguish which nodes belong to which dataset. The different colours indicate that the nodes have different rdf:types (e.g. light blue being a gene and dark blue a “Disease, Disorder or Finding”). However, not all data was colour-labelled by GraphDB, probably due to the use of a federated query where some data might not have been automatically processable for these functionalities. (b) Graph2VR visualisation using the hierarchical view as layout (not the class hierarchy). Comparison of data visualisations of GraphDB and Graph2VR.

DisGeNET is a resource of gene–disease associations (GDAs) [32]. It offers a SPARQL Endpoint that allows users to query for those associations. Known variations in the gene sequence of the actual patient data can be compared with known GDAs from the database to see which of the patient’s phenotypes match phenotypes known to be associated with the gene carrying the variant. In this way, we can create “closed loops.” These loops start with the patient, their phenotypes, and the genes associated with their variant’s transcripts. Within DisGeNET, “any” Disease, Disorder, or Finding that is associated with the patient’s phenotypes is retrieved, together with any GDAs linked to it. From here, the loop is closed by connecting the GDAs from DisGeNET to the genes associated with a patient. Any GDAs that cannot form a closed loop are not included in the graph. Additionally, any evidence (source) is included where available. Building such a loop can help users interpret these data and pinpoint the causal variant in the patient in order to provide them a genetic diagnosis. Figure 7 shows different visualizations of this data.

### Use case 2: Evaluation

For scenario 1, we ultimately found that the user did not experience added benefit from using VR over a conventional 2D visualization because of the small number of nodes that could also be easily displayed and navigated in 2D. In retrospect, we should have filtered the original dataset less or not at all so that it would have been larger and necessitate the use of 3D in VR. Alternatively, we could also have expanded the graph with additional data sources to increase its size and complexity. In scenario 2, VR became much more useful because the networks became complex and interconnected. Here, the 3D VR representation allows the user to find nodes more easily because they are spaced out much farther than is possible in 2D, while relationships are also easily explored by grabbing and moving nodes along with their edges. We hypothesize that visualizing relationships between these different layers of genotypic and phenotypic information can accelerate the prioritization of disease-causing DNA variants. Lastly, in scenario 3, a manual 2D layout for one patient is insightful, but when scaling up to multiple patients and working with automated layouts, VR offers much more freedom and interactivity to explore these rich and complicated networks, especially as it allows users to expand specific predicates with more precision. This ability to expand the graph using multiple different endpoints is a feature of Graph2VR that not many comparable tools (like Relfinder) offer [33].

### Use case 3: PubChem

#### Use case 3: background

PubChem and Drugbank are two large databases about chemical substances and drugs. The PubChem website describes itself as “the world’s largest collection of freely accessible chemical information,” whereas Drugbank is a “knowledge base of about 500 000+ drugs and drug products” [6, 34, 35]. Thus, while Drugbank contains data about drugs and their attributes, PubChem contains chemical information. Both datasets, but especially PubChem, also provide good examples of larger datasets. There is also overlap between them, and in many cases there are references from one dataset to the other. If this is not (yet) the case, identifiers like Chemical Abstracts Service (CAS) numbers might be considered for matching. We chose this example with the idea that large graph databases may impact the real-time user experience in Graph2VR. Besides testing the handling of such large datasets, the goal of this scenario was to find out whether it is possible to use Graph2VR to enrich data about ATC codes with additional chemical information from PubChem, using Drugbank as an intermediate database.

#### Use case 3: Scenarios

The context of the scenario is to combine Drugbank, PubChem, and Dutch ATC codes to enable users to understand how drug names might match underlying chemical compounds and their properties.

ATC codes classify drugs not only by their chemical components, like aspirin, but also by how they are applied (whether it is a cream, an injection, or a pill) and what the drug is used for. Most drugs have only one ATC code, but this can vary. Cortisone, for example, has more than 10 different ATC codes. During the COVID-19 pandemic in 2020, the Lifelines cohort study collected data from participants across the northern Netherlands. On 30 March 2020, a Lifelines cohort study started to identify protective and risk factors for SARS-CoV-2 susceptibility [36, 37]. They sent out online questionnaires to capture several factors, including which drugs participants used. For this, our group developed a method to semiautomatically map participants’ free-text answers about the drugs to ATC codes. We created an ontology that referred from ATC codes to Dutch drug names to support experts in mapping those drugs to standardized codes faster. This would help to standardize and then effectively filter datasets based on the groups within the ATC hierarchy. This ATC standardization enables statistical evaluation to identify protective or risk factors based on certain groups of medications. The ATC code Ontology contains Dutch drugs with different names, but those are related to ATC codes within an English ATC code ontology [36]. In this use case, we wanted to see whether this ontology could be enriched with information from Drugbank or PubChem. The URIs used in the ontology were created generically based on a hash function. Those URIs will probably not match any common URL standard, but the ATC codes themselves can be used to connect the ontology to other drugs.

The focus here was to start with an existing entry, which could be the ATC code of a Dutch drug, and see what medical and chemical information can be added. For example, there are dozens of drugs that actually contain aspirin, and these could be treated as one drug for analysis (depending on the question), but this requires that users are able to resolve this information.

#### Use case 3: methods and materials datasets used

We used the following datasets:

- “Dutch ATC code Ontology” is a collection of Dutch drug names put together for semiautomatic mapping and connected to their respective ATC codes. The dataset is structured by the ATC code ontology [36].
- “Drugbank” contains information about drugs, including names, different IDs, and targets. It is available for academic research [6].
- “PubChem” holds general information about almost all registered small chemical compounds. It is available as RDF [7, 38].

##### RDF preparation

To execute this query, it is necessary to allow insert statements, which can be done with the isql command before running the actual insert query. grant execute on DB.DBA.SPARQL_INSERT_DICT_CONTENT to “SPARQL”;

##### Graph2VR application

We prepared Graph2VR by entering the address of our local Virtuoso instance with Drugbank and PubChem in separate instances of Virtuoso. As recommended, we uploaded each of PubChem’s graphs (substances, compounds, synonyms, etc.) into separate graphs in Virtuoso. It is not necessary to load the whole dataset, just the relevant graph(s).

##### Specific example queries/challenges

In this example, data conversion is not the issue. PubChem already offers its data as Linked Data, and a script for the conversion into RDF is readily available for Drugbank. We used version5.1.10 of Drugbank (April 2023) and converted it to n-triples. To do so, we reused a script from the Bio2RDF repository [39–41]. The script translated the .xml file from Drugbank into n-triples, which were then uploaded to a Virtuoso server. One particular issue with these two datasets compared to those in the previous use cases is their size. At 7 GB, Drugbank is too big to be uploaded into Virtuoso via the web interface, so we had to use the bulk loader to load the data into the Virtuoso server [9]. While the Drugbank import was relatively swift, the PubChem dataset occupied nearly 1TB (938 GB) of storage once imported into Virtuoso. In addition, some known issues with Virtuoso 7.x caused memory leaks. This issue can be avoided by using an older version of Virtuoso (e.g. version 6.1) [42]. It took almost 3 days to import all of PubChem’s graphs into a Virtuoso server using the bulk loader. As mentioned, PubChem is divided into several graphs, and selecting only the relevant ones will save time and storage. We imported into Virtuoso following the guidelines provided on the PubChem website [7]. Based on the structure of PubChem (see Table 1 of [38]), we know that the relevant information to connect substances in PubChem with their respective counterparts in Drugbank is connected via the predicate sio:has-attribute to a depositor, which then has a value (sio:hasValue) and a type. The prefix “sio” stands for the Semanticscience integrated ontology (SIO) and is a shortcut for “http://semanticscience.org/resource/” [43]. The type is encoded with CHEMINF 000406 for the DrugID, which refers directly to the ID within Drugbank. Alternatively, CHEMINF 000446 is the type for the CAS number, which is a unique classifier for each chemical substance in the CAS Registry. The prefix “CHEMINF” stands for Chemical Information Ontology and is an abbreviation for “http://semanticscience.org/resource/” [44].

For PubChem, such a SPARQL CONSTRUCT query could look like this:
PREFIX sio: <http://semanticscience.org/resource/>
PREFIX rdf: <http://www.w3.org/1999/02/22-rdf-syntax-ns#> PREFIX cheminf: <http://semanticscience.org/resource/>
PREFIX compound: <http://rdf.ncbi.nlm.nih.gov/pubchem/compound/>
Construct{
    ?depositor_identyfier sio:SIO_000011 ?compound.
    ?depositor_identyfier sio:SIO_000300 ?DrugID.
    ?depositor_identyfier rdf:type cheminf:CHEMINF_000406.
    ?depositor_identyfier2 sio:SIO_000011 ?compound.
    ?depositor_identyfier2 sio:SIO_000300 ?CASno.
    ?depositor_identyfier2 rdf:type cheminf:CHEMINF_000446.}
WHERE {
   ?depositor_identyfier sio:SIO_000011 ?compound.
    ?depositor_identyfier sio:SIO_000300 ?DrugID.
   ?depositor_identyfier rdf:type cheminf:CHEMINF_000406.
OPTIONAL{
    ?depositor_identyfier2 sio:SIO_000011 ?compound.
    ?depositor_identyfier2 sio:SIO_000300 ?CASno.
    ?depositor_identyfier2 rdf:type cheminf:CHEMINF_000446.
}
} ORDER BY (?DrugID) Limit 10

PubChem uses the URIs for the compounds or substances to refer to the actual substances, but some depositors do describe the relations to identifiers. In this case, those are the SIO codes sio:SIO_000011 (”is attribute of”) that connect those references with the actual compound, the predicate sio:SIO_000300 (”has value of”) that actually holds the identifier, and an identifier that the node is linking to, in this case cheminf:CHEMINF_000406 (”Drugbank identifier”) and cheminf:CHEMINF_00446 (”CASNO”) to describe the CAS number of a compound. Since PubChem has information about 30 000 Drugs, we limited the number of results to 10. Otherwise, we would probably run into a timeout.

In the rdf version of Drugbank that we created with the Bio2RDF scripts, the ATC codes are related to each drug using the following schema:

<http://bio2rdf.org/drugbank:DB00945>

<http://bio2rdf.org/drugbank_vocabulary:x-atc>

<http://bio2rdf.org/atc:B01AC06>.

This triple encodes aspirin with the Drugbank ID “DB00945” has the ATC code “B01AC06.”

The third data source, the Dutch ATC code ontology represents ATC codes as URIs, as well as the Drugbank representation. Since there is no reference between the two and their URIs differ (although they should be the same), we generated the relation, which made navigation between the graphs in Graph2VR easier later on. The only difference between the two is the prefix of each database, which is followed by the actual ATC code. Since both define ATC codes, we can use the owl:sameAs relation to connect them.

PREFIX uatc: <http://purl.bioontology.org/ontology/UATC/>
PREFIX owl: <http://www.w3.org/2002/07/owl#>
PREFIX atc: <http://bio2rdf.org/atc:>
INSERT {
GRAPH <http://localhost:8896/ATC> {
?uatcUri owl:sameAs ?atcUri.
}
}
WHERE {
?uatcUri rdf:type owl:Class.
FILTER(STRSTARTS(STR(?uatcUri), “http://purl.bioontology.org/ontology/UATC/”)) BIND(URI(CONCAT(“http://bio2rdf.org/atc:,” REPLACE(STR(?uatcUri), “http://purl.bioontology.org/ontology/UATC/”, ““))) AS ?atcUri)
}

As a starting query, we wanted to combine all three databases. Therefore, this federated SPARQL query was added to the settings file to serve as starting query. In this case, the databases are hosted on different local instances of Virtuoso. However, for the navigation with Graph2VR, it would have been easier to put all of them in one server but in different graphs. In the following example PubChem, the local Virtuoso server with the PubChem database is accessible on port 8890, Drugbank on 8891, and the ATC code ontology on port 8896. (We limit the number of results to 10 again, since the number of results would be overwhelming in Graph2VR given that it could even handle so many nodes, and the query would most likely run into a timeout.)

PREFIX sio: <http://semanticscience.org/resource/>
PREFIX rdf: <http://www.w3.org/1999/02/22-rdf-syntax-ns#>
PREFIX cheminf: <http://semanticscience.org/resource/>
PREFIX drugbank: <http://bio2rdf.org/drugbank_vocabulary:>
PREFIX bio2rdf: <http://bio2rdf.org/bio2rdf_vocabulary:>
PREFIX xsd: <http://www.w3.org/2001/XMLSchema#>
PREFIX uatc: <http://purl.bioontology.org/ontology/UATC/>
PREFIX owl: <http://www.w3.org/2002/07/owl#>
PREFIX rdfs: <http://www.w3.org/2000/01/rdf-schema#>
CONSTRUCT {
?drugbankURI bio2rdf:identifier ?DrugbankID.
?drugbankURI drugbank:x-atc ?atcUri.
?drugbankURI rdfs:label ?label.
?uatcUri owl:sameAs ?atcUri.
?compound sio:SIO_000011 ?depositor_identifier.
?depositor_identifier sio:SIO_000300 ?DrugbankID.
?depositor_identifier2 sio:SIO_000011 ?compound.
?depositor_identifier2 sio:SIO_000300 ?CASString.
}
WHERE {
SERVICE <http://localhost:8891/sparql> {
?drugbankURI bio2rdf:identifier ?x.
?drugbankURI drugbank:x-atc ?atcUri.
?drugbankURI rdfs:label ?label. BIND(str(?x) AS ?DrugbankID)
FILTER (str(?DrugbankID) = “DB00945”).
}
SERVICE <http://localhost:8896/sparql> {
?uatcUri owl:sameAs ?atcUri.
}
SERVICE <http://localhost:8890/sparql> {
?depositor_identifier sio:SIO_000011 ?compound.
?depositor_identifier sio:SIO_000300 ?DrugbankID.
?depositor_identifier rdf:type cheminf:CHEMINF_000406. OPTIONAL {
?depositor_identifier2 sio:SIO_000011 ?compound.
?depositor_identifier2 sio:SIO_000300 ?CASString.
?depositor_identifier2 rdf:type cheminf:CHEMINF_000446.
}
}
}
LIMIT 10

#### Use case 3: Results

**Figure 8.**
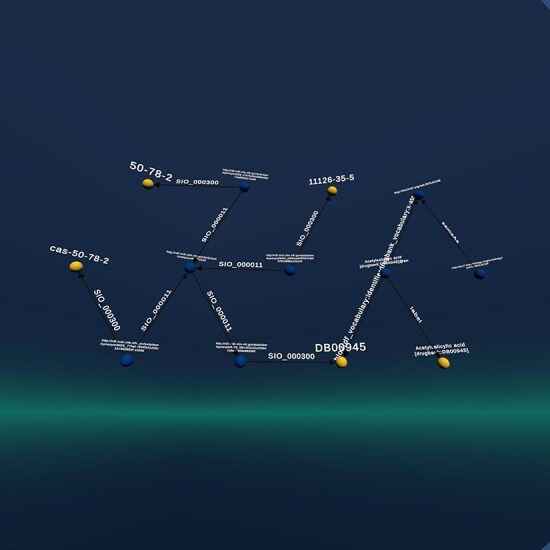
The graph resulting from the query is based on all three graphs. It connects the entries about the ATC codes of aspirin from Drugbank with the ATC code ontology, connects the Drugbank entry for aspirin to PubChem, and displays its CAS number(s).

The result of this query is a graph that contains information about aspirin but extracts this information from all three databases (see Fig. 8). In Graph2VR, we can now start to navigate and expand the resulting graph. Depending on which database is required to deliver additional information, it is possible to switch between the databases and to open new connections or start querying. Having the databases on one server would allow us to navigate the graph without having to select the database, even though we could do that by selecting the respective graph. However, as PubChem is so large, queries often time-out or take a long time, so having the databases in separate Virtuoso instances or selecting the respective graph can speed the process up. Once we have that graph in Graph2VR, further exploration can start. The default timeout for Virtuoso is 20 s; this can be increased. However, PubChem is so large that queries can take hours, which is not feasible in a VR application, where results should be displayed almost instantly.

**Figure 9.**
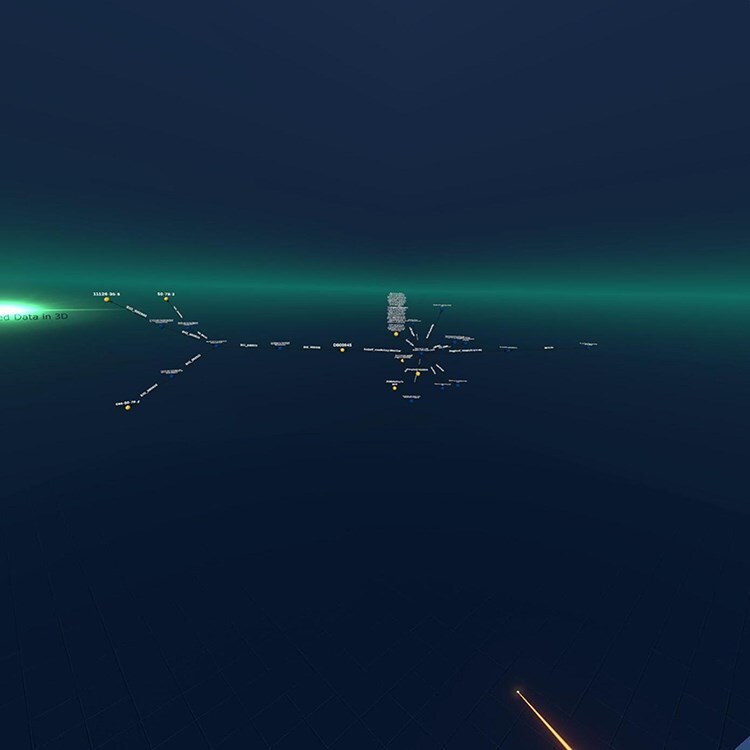
The graph from the previous query about Aspirin. It has been expanded using data from Drugbank and stretched manually. At left are the CAS numbers from PubChem, in the middle the expanded Drugbank entry, and on the right the ATC code from the third database, demonstrating that this can be used as a starting point in Graph2VR.

In Fig. 9, the PubChem parts of the graph have been pinned down on the left side, the ATC code Ontology is pinned to the right, and the Drugbank part in the middle has been expanded with additional knowledge.

#### Use case 3: Evaluation

Use case 3 was difficult to execute for multiple reasons. First, the available query endpoints for PubChem and Drugbank are only offered by third parties, and their data versions are about 10 years old. Even if there was an up-to-date endpoint for PubChem, it would probably be too slow for querying due to the high volumes of data. Second, while the RDF resources are up-to-date, they are not trivial to use because they must be imported into a local triple store before querying. Even then, queries must be cleverly constructed to ensure execution in a reasonable time-frame (seconds to minutes rather than hours to days). Third, even if we manage to get relevant query results into Graph2VR, the user might still be confused about some node and edge values, such as “SIO_000300” and “CHEMINF_00446.” Those CUIs are not very intuitive because most people will not immediately know the meaning of the machine-readable CUIs employed by the different databases. To replace these with more human-friendly labels like “has value” and “has CAS Registry Number,” we must import additional ontologies, such as SIO and CHEMINF [43, 44]. After we dealt with these issues, Graph2VR was able to effectively navigate, expand, and visualize these data. In particular, Graph2VR’s ability to expand the graph using multiple different endpoints was instrumental in implementing this use case.

## Conclusion

From the three use cases, we learned several lessons:

- Complex data that features many layers and dimensions benefits greatly from the use of VR, as compared to traditional 2D visualizations, because VR offers endless space to order the data and fast navigation tools such as teleportation.
- Expanding and exploring a graph in Graph2VR can be much faster than writing SPARQL queries. The relevant predicates can often be identified simply by looking at the predicate’s label in the menu in Graph2VR.
- Often, multiple different endpoints are used when exploring data, which is something Graph2VR can do, in contrast to most comparable tools.
- Nodes and edges often contain only machine-readable labels that must first be replaced with additional ontologies before a human can make intuitive sense of the graphs.
- Very large data sources can be problematic, regardless of remote or local query execution. To make use of them, optimized imports and smart queries must be designed first.

Based on these initial findings and the mainstreaming of VR headsets, we believe that it holds much potential for research. However, there are many opportunities and challenges ahead, which we discuss below.

## Discussion

In this section, we first delineate the pressing challenges we faced during this pilot study and then discuss opportunities for future development.

### Scalability issues limit direct use of big Linked Data

**Figure 10.**
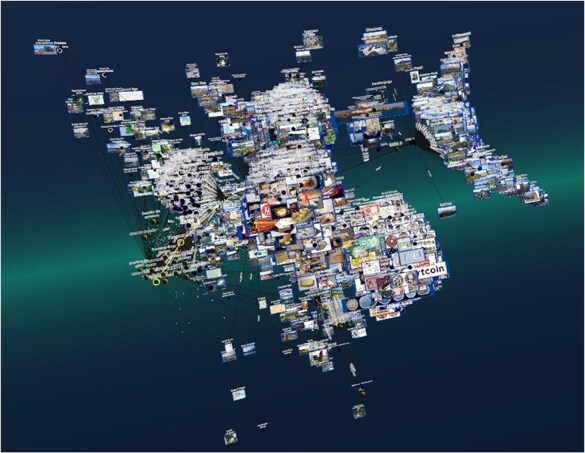
Example of a large graph demonstrating the rendering capabilities of Graph2VR on an RTX 4090. It shows about 17 000 nodes handled by Graph2VR. A framerate drop to 14 fps was observed during the layout phase (the fps increase significantly afterwards). Dataset: DBpedia.

In the last use case, we faced major challenges related to the size of the data and the limitations of Semantic Web technology and VR rendering. What we learned from the Drugbank use case is that SPARQL Endpoints need to respond relatively fast for a seamless Graph2VR experience. Timeouts cannot be indicated in Graph2VR, so the user does not immediately know whether a delay indicates a timeout, a bug, or a connection issue. In addition, very large graphs reduce the framerate of the headset. We found that we reached the limit when rendering 5000 nodes on a GTX 1060 or 17 000 nodes on the RTX 4090 with 14 fps during layouting. An example of such a large graph including images is shown in Fig. 10.

### Disconnect between user mental concept and graph structure

In addition to scaling issues, we noticed that it often took users quite some time to internalise the Semantic Web structure. In these cases, the concepts and relationships that the user expected to see did not match the knowledge graph provided. These could be small issues, such as labels being different than expected, or bigger issues where relationships that users expected to be direct actually required intermediate steps in the knowledge graph. For example, in Use case 1, the user(s) expected variables to be directly linked to cohort studies. In actual fact, however, they are grouped either by datasets, collection events, or subpopulations. Therefore, in Use case 2, we had to invest time in creating a small application ontology to make sure the data were projected in a knowledge graph that suited the scenarios. An alternative solution might be tooling to help users understand the knowledge graph, as was done in Relfinder (no longer accessible since the end-of-life of Adobe Flashplayer), which enabled users to easily find a path given a starting and ending node.

### Representativeness of the use cases

A huge topic of debate between the study participants was whether our initial findings could be generalized to other life science use cases. We selected our use cases to provide diversity in complexity and scale and in the level of refined semantic annotation and to range from molecular detail to high-level general concepts. Additionally, we have done a short survey of the available literature on the use of Linked Data and/or VR technology in life sciences. For example, when analysing Bioportal categories, the ontologies cover health, organisms, different levels of molecular data, and imaging [45]. In addition, we explored emerging uses of VR via a PubMed search for “virtual reality” (over 20 000 hits). When analyzing this for research applications, we observed an over-representation of studies involving “surgical,” “molecular,” “anatomical,” and “imaging” analysis applications. We speculate that our use cases are representative of many of the ontologies listed. However, we are curious how Graph2VR performs when applied to very specific use cases that we did not cover, such as microscopy and imaging. Therefore, we hope to achieve future funding to run a new experiment to assess the tool in a more systematic way, choosing the users that do the evaluation with some specific background and identifying the required knowledge that the users must have to run those experiments and creating a proper evaluation of the performance of the users in those experiments when doing specific tasks.

### Envisioning future visual enhancements

During the use case experiments, there were many instances where we discussed the potential added value of visual cues to enhance the experience. In the current Graph2VR, we use a standardized panel of visualizations for the nodes and edges. While we think this generality (i.e. not hard-coding shapes and colours based on assumptions on the RDF representation) is the power of Graph2VR, there are ideas about how to enable user annotation to influence the rendering. In our previous Graph2VR paper, we discussed additional options for the use of colors, shapes, and layouts [1]. We believe a first layer of enhancements would be to allow the user to add a knowledge graph that details how different elements should be visualized. For example, based on class or property differences, the user could specify what colors could be applied or even what shapes could be used, e.g. to find patterns in the data quickly.

For the long-term future, we dream of mixing the knowledge graph with 3D representations of real-world artifacts. For example, users could be enabled to create a view in Graph2VR that shows a map of the brain with Linked Data about the phenotypes, diseases and genes/variants known to be associated with that part of the brain. Similar projects (without interactive SPARQL capabilities) already exist [46].

## Future work

While we believe Graph2VR has been a good tool to explore the potential of combining VR and the Semantic Web for scientific use cases, we encountered many areas in which we believe it can be improved. A public list of feature requests is available at https://github.com/molgenis/Graph2VR/issues. Below, we summarize the most important suggestions for the future, hoping for community improvement of this open-source tool.

### Improving the search function

The search function in Graph2VR is currently rather limited. Because we could not make assumptions about the availability of optimized search indexes available in the source, we based the search on either a regex search or, if available, a bif:contains full-text search [47]. The snippets below show the regex query and bif:contains query, respectively. (Please note that this is an expert from Graph2VR’s C# code for generating a SPARQL query, not a valid SPARQL query):

select distinct {variableNode.GetQueryLabel()} AS ?uri (SAMPLE(?name) AS ?name) where {{
{variableNode.graph.GetTriplesString()}
{variableNode.GetQueryLabel()} rdfs:label ?name.
?uri(ˆ(<>| !<>) | rdfs:label | skos:altLabel) ?entity. BIND(STR(?entity) AS ?name).
FILTER REGEX(?name, ‘{searchTerm}’, ‘i’).
}}
LIMIT {searchResultsLimit}”;
select distinct {variableNode.GetQueryLabel()} AS ?uri (SAMPLE(?name) AS ?name) where {{
{variableNode.graph.GetTriplesString()}
{variableNode.GetQueryLabel()} rdfs:label ?name.
?name bif:contains ““‘{AddStar(searchTerm)}’““.
}}
LIMIT {searchResultsLimit}”;

For the search function, we adapted SPARQL queries from a paper on “Efficient and Effective SPARQL Autocompletion on Very Large Knowledge Graphs” [48]. However, the results of this search function often do not match the intended search, e.g. when searching on DBpedia for “Donald,” one would expect results like “Donald Duck,” but the first result we got was “Amy McDonald.” This result is factually not wrong, but there are often many such irrelevant results. We are confident that there are more effective ways to search, and a better search function would make navigating the database much more straightforward. We did not invest much time in implementing a better search functionality, even though the application would clearly benefit. An instance of advanced search functionality for Linked Data is demonstrated by the commercial tool Metaphactory [49]. The fact that Graph2VR also operates on external databases makes it more challenging to implement a good search because we cannot simply upload the whole database to a local SOLR or Elastic search server, but rather have to limit ourselves to the options that SPARQL queries offer [50, 51].

### Improving query options

In the current release of Graph2VR, the user has several options for building queries and interacting with the data. Users can expand, collapse, and build queries; add nodes and edges; turn nodes into variables; and define optional triples, limits, and ordering. However, not all SPARQL commands have been implemented, even though the DotNetRDF engine Graph2VR uses would support them and the graphs could be displayed. We would like the GUI to expose more of these functions, especially nested queries, filters, offsets, and recursive requests, e.g. to iterate opening the rdfs:subClassOf predicates upwards to owl:thing. For example, for Use case 3, we had to create a starter query that used a nested query to combine data from the three data sources using the bind option to align mismatched ATC code URIs (PubChem, ATC, Drugbank). Ideally, we would like to enable the user to perform this interactively through the user interface.

### Multi-player VR

During the use case evaluation, only one participant at a time could experience VR. This forced us to evaluate the added value of VR versus the 2D representation. The collaborators on the use cases took turns being the VR controller. While this worked surprisingly well, it greatly limited the potential for interaction between participants. We all experienced that being submerged in VR makes it easier and more natural to work with and navigate large/multiple graphs as the screen feels 360 degrees instead of only a small square window. An obvious future enhancement would thus be enabling multiple users to work in the same VR. We believe that adding other VR headsets as observers would be relatively easy; however, having multiple actors with controls and interaction at the same time might require a fundamental rewrite of the software.

### Augmented Reality

In the current version of Graph2VR, the user is in an endless world with a small platform that is a visual anchor in the virtual environment. It would be interesting to project the graph in the real world instead, especially in combination with a multiplayer feature where multiple people can interact with the graph(s) together. There are already similar projects with the Microsoft Hololens, but as far as we have seen, these are more for visualization of network graphs and 3D models and less for interaction with Linked Data [46]. That tool actually provides gesture and voice controls.

### Safety and Security

While both critical in software systems, safety and security address different aspects of system integrity. Safety ensures the system’s reliability and correct function, whereas security protects against external threats and unauthorized access.

Safety: We know that Graph2VR can and probably will crash when the GPU is overwhelmed with too many nodes. When the application crashes, we might like to continue from a restored session from shortly before it crashed. Therefore, an auto-save function, e.g. saved just before sending a query, could be used to prevent data loss. Data loss in this case means only loss of temporary graph(s) in Graph2VR, as Graph2VR does not write anything back to the database. Security: In terms of security, Graph2VR needs some improvement. It can send SPARQL queries to SPARQL Endpoints, but it does not yet support using a login (username and password). Therefore it requires the database to be open. For linked open data like DBpedia or any public SPARQL endpoints, this is not an issue. But when a Virtuoso Server is set up locally, its SPARQL Endpoint is, by default, accessible to anyone on the local network. When working with sensitive data, a tool that does not support login is problematic. We dealt with this by using a distinct, separate network to which only the relevant devices had access, rather than a shared network.

### Enable combining endpoints

Currently, Graph2VR users cannot create a query graph that involves multiple RDF sources at once (e.g. combining PubChem, ATC, and Drugbank), while the users intuitively expect to be able to do so. Therefore, to be able to use different endpoints, it would be a useful feature to see the overlap between the nodes that are present in the graph and those that are also part of the second database. The necessity to preidentify nodes present in two separate data sources, or the process of trial and error, significantly limits usability, especially in a tool meant to be used for exploration. It might be helpful if it would display nodes that are not present in the current Endpoint as transparent, so that users can immediately see where connection points are to be expected. This might be quite extensive and lead to the necessity to request the presence of each node in the other database. For sensitive datasets like that in the VIP use case, this would be unwanted.

### SPARQL query logging

While the Graph2VR GUI does much of the heavy lifting in querying, there are instances where it would be useful for the user to be able to see the SPARQL query that has actually been executed. Such a feature would aid not only in debugging but also in verifying queries and ensuring documentation and reproducibility. It could also be useful to be able to display the current query within the application to see what the selected patterns and options translated to. Exporting and storing SPARQL queries created in Graph2VR should be easy to implement, but this has not yet been done.

### Shivering graph

Graph2VR tends to be intuitive for the exploration of graph databases. However, one issue that we faced regularly was that the graph often began to increasingly “shiver” or jitter over time while it was grabbed. The reason for this seems to be the angle between the controllers, the center of the graph, and a reference point. The center is recalculated regularly, as is the reference point. The further these three points are from each other, the more the graph begins to shiver. The reference point is also affected by the layout algorithms. This might be a floating point/rounding error and is something that should be improved upon.

### Preserving save state

During our improvements of Graph2VR, we often created new versions of the tool. To install an update on the headset, the old version of Graph2VR has to be removed. Uninstalling the application also removes its save states due to the way the Quest handles uninstalling applications. We therefore recommend that users manually back up the save states before removing Graph2VR from the headset. This backup can be performed using Sidequest.

### Compatibility of Graph2VR with different VR headsets

Graph2VR was designed to be compatible with the Vive and Quest2/Quest3 headsets. The OpenXR toolkit that we used to bind the controls checks for the headset to set the bindings and adjusts the angle of the laser [52]. Currently, there is no specific support for additional headsets. If we had used SteamVR instead of OpenXR, many headsets would have been supported out of the box. We switched to OpenXR mainly because it allowed us to build a stand-alone version of the application on the headset.

### Compatibility of Graph2VR with different graph databases

Graph2VR was tested with Virtuoso servers like DBpedia and local Virtuoso servers. Initially, we thought, DotNetRDF would also support other databases like GraphDB, Allegro Graph, and more [53]. In principle, it does, but it seems to use different functions to talk to the different Endpoints. Currently, we have implemented only one of them. However, it should not be too difficult to add support for additional types of databases.

### Improved colors

In terms of color, Graph2VR largely follows the VOWL schema, although it has not been fully implemented and adjustments were necessary for the 3D representation [54]. Whether this color scheme is the best choice remains a point of discussion. Other tools such as Gruff or GraphDB, use different colors to code for different types of nodes [31]. This approach allows easy visual distinction of different types of nodes, which can be useful, as in Figs 5, 6, and 7. It is already possible to adjust the colours in the settings file, but this could be made even more flexible by specifying the predicate or type and the color the entities should have.

### Layouts

One major advantage of VR over 2D layouts is that it is much harder to run out of space. Graph2VR already offers four different layout algorithms, but there could be more. Those could use other criteria, predicates, and algorithms to restructure the data. During the user evaluation, some users specifically asked for a layout that would situate the data around the user, e.g. projecting the data on the inside of a large sphere or the ability to enter a subgraph like in the semantic bubble chart [55].

### Combining Graphs

In some situations, it would be helpful to merge different graphs in Graph2VR. We have already added this as a feature for queries, i.e. to either have a stack of result graphs or to combine them into a single graph. However, it would be useful to be able to do this with two independent graphs already present in Graph2VR. Users would expect that, once they add an edge, they could visually merge the two graphs and handle them as one, and that layout algorithms could be applied to that merged graph. This involves a design choice how to handle nodes with the same URI present in the two graphs while merging: (I) Merging all nodes with the same URI to one node, or (II) keeping them separate and allowing duplicates in a graph, which could interfere with other parts of the codes like expanding the graph.

### Bridging points between different endpoints

When working with multiple SPARQL Endpoints in the same Graph2VR session, it might be useful to be able to see which of the visible nodes are also present in the other endpoint. Upon switching databases, there is no immediate way to determine which of the present nodes exist in the new database, i.e. which ones can potentially serve as connectors for further expansion and exploration. The idea of automatically verifying which nodes are present in another SPARQL Endpoint is appealing. Nodes that are not present in the server might be displayed as semitransparent. We decided not to do this automatically because the user might not want to share that data, for various reasons, like having a slow database or not wanting to send too many queries or sensitive information. It should be up to the user to determine whether to share the current graph’s content for finding the overlapping nodes.

## Supplementary Material

baaf008_Supp

## Data Availability

This article is based on Graph2VR software version 1.2.6, see GitHub for version history https://github. com/molgenis/Graph2VR [1] The usage of Graph2VR is explained in the user manual [10]. The software/source code of Graph2VR is open source under the permissive open-source license LGPLv3 and includes thirdparty software that is available under its own license) We welcome contributions and suggestions for improvement at Github: https://github.com/molgenis/Graph2VR/issues A Graph2VR tutorial playlist is available on YouTube [56]: https://www.youtube.com/playlist?list=PLRQCsKSUyhNIdUzBNRTmE-_JmuiOEZbdH The data used in this article are available online. For use case 1, we added a snapshot of the Molgenis catalogue data as it was when we used it, see supplementary file catalogue-demo.rdf. For use case 2, all data and scripts are available at https://zenodo.org/doi/10.5281/zenodo. 10 848 352. For use case 3, the ATC code ontology is available via https://github.com/AJKellmann/Scriptsfor-the-translation-of-medicine-usage-data-to-ATC-codes/tree/main. The PubChem and Drugbank data can be obtained from Drugbank https://go.drugbank.com and PubChem https://ftp.ncbi.nlm.nih.gov/pubchem/RDF/ directly.
