# Supplementary material for: An exploratory study combining Virtual Reality and Semantic Web for life science research using Graph2VR
Source: Database (Oxford). 2025 May 20;2025:baaf008. doi: 10.1093/database/baaf008 (PMC12090995; doi:10.1093/database/baaf008)
Supplement: baaf008_Supp [file baaf008_supp.zip › suppl_data/Attachments.pdf]

8. Attachments

Appendix A. Supplementary information Methods

Appendix A.1. Supplementary screenshot sidequest

The following screenshot of Sidequest shows the path to the Graph2VR folder on Quest 2. This is also the path that needs to be saved for a backup or to place a settings file for the standalone version.

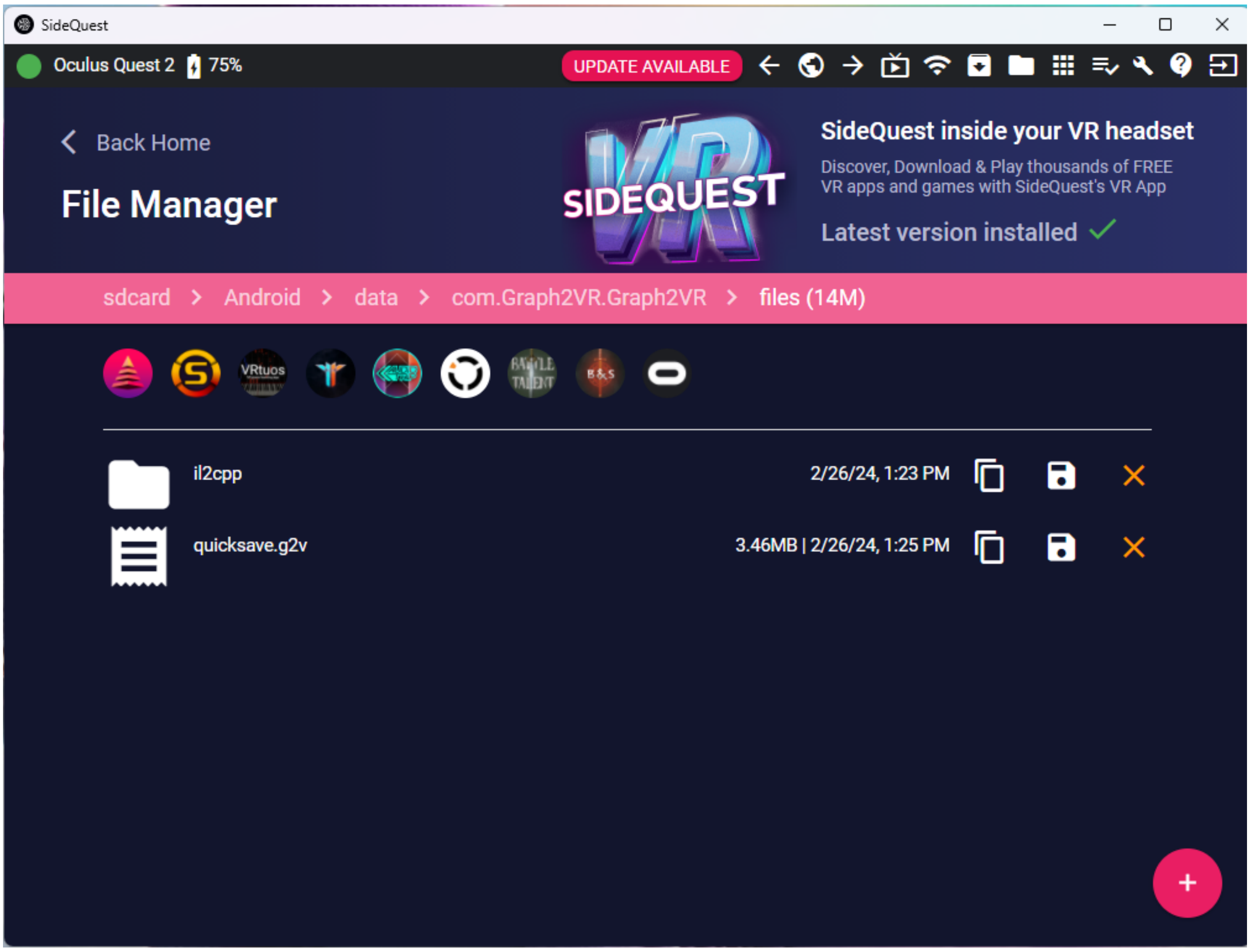

Figure A.11: Sidequest showing the Graph2VR folder with the quicksave save file.

Appendix B. Supplementary information Use case 1

Appendix B.1. Supplementary screenshot Molgenis Catalogue class hierarchy in Protégé

We extracted the data from MOLGENIS catalogue `https://molgeniscatalogue.org` using the /api/rdf endpoint. The resulting class structure is listed in Figure B.12 below.

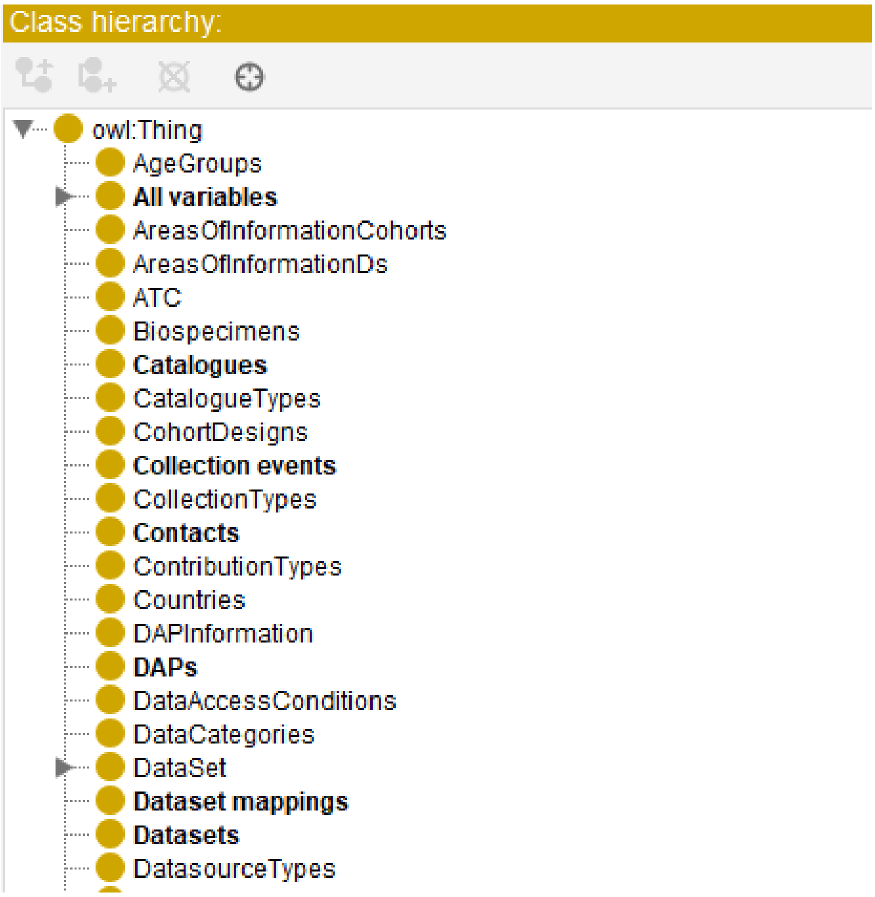

Figure B.12: A screenshot of Molgenis Catalogue's class hierarchy in Protégé.

Appendix C. Supplementary information Use case 2

Appendix C.1. Namespaces used

Below an overview of the namespaces used in use case 2.

**Prefix**	**Namespace**	**Usage**
rdf	http://www.w3.org/1999/02/22-rdf-syntax-ns#	Generic rdf namespace
rdfs	http://www.w3.org/2000/01/rdf-schema#	Generic rdfs namespace
dcterms	http://purl.org/dc/terms/	Description predicate for longer name than rdfs:label where needed
obo	http://purl.obolibrary.org/obo/	Sequence ontology (SO_<number>) & HPO (HP_<number>) IRIs
sio	http://semanticscience.org/resource/	All kinds of IRIs to describe data (predicates & rdf:type objects)
ncbigene	http://identifiers.org/ncbigene/	NCBI Gene ID IRIs
refseq	http://identifiers.org/refseq/	NCBI Reference Sequences IRIs
ensembl	http://ensembl.org/glossary/	Using the Ensembl glossary, String-formatted VEP data in the VIP output is converted to their corresponding semantic web IRIs
vip	urn:uuid:E4CBEA11-46B8-4B68-A202-B9FC8E5BE255#	General IRIs specific for this project
	urn:uuid:[output file specific UUID]#	Patients/variants/transcripts specific for a single generated turtle output file

Table C.1: All namespaces with their prefix and how they were used within this use case.

Appendix C.2. The Full Dataset for use case 2

When we uploaded the whole VIP dataset into Virtuoso and displayed the whole graph in Graph2VR, the first graph shown was relatively unordered and hard to understand. However, after adding only a few subclass relations and using the class hierarchy layout, a much more structured layout was formed (see Figure C.13). Its base structure is built up based on the class hierarchy, using the rdfs:subclassOf property, and building a horizontal tree structure where individuals, each encoded by the rdf:type property, are stacked on top of each other beneath their class. The third dimension is then used for attributes of the individuals. Nodes that do not follow this class hierarchy pattern, i.e. do not have an rdf:type or rdfs:subclassOf property and are not connected to such a node, cluster somewhere at the bottom of the graph. This graph gives an overview of our (rather small) dataset as a whole. Displaying it in other applications like GraphDB will result in hairballs of nodes and edges that are hardly readable. The advantage of VR is that we are not spatially limited and can actually get close enough to see and read what is represented in the graph.

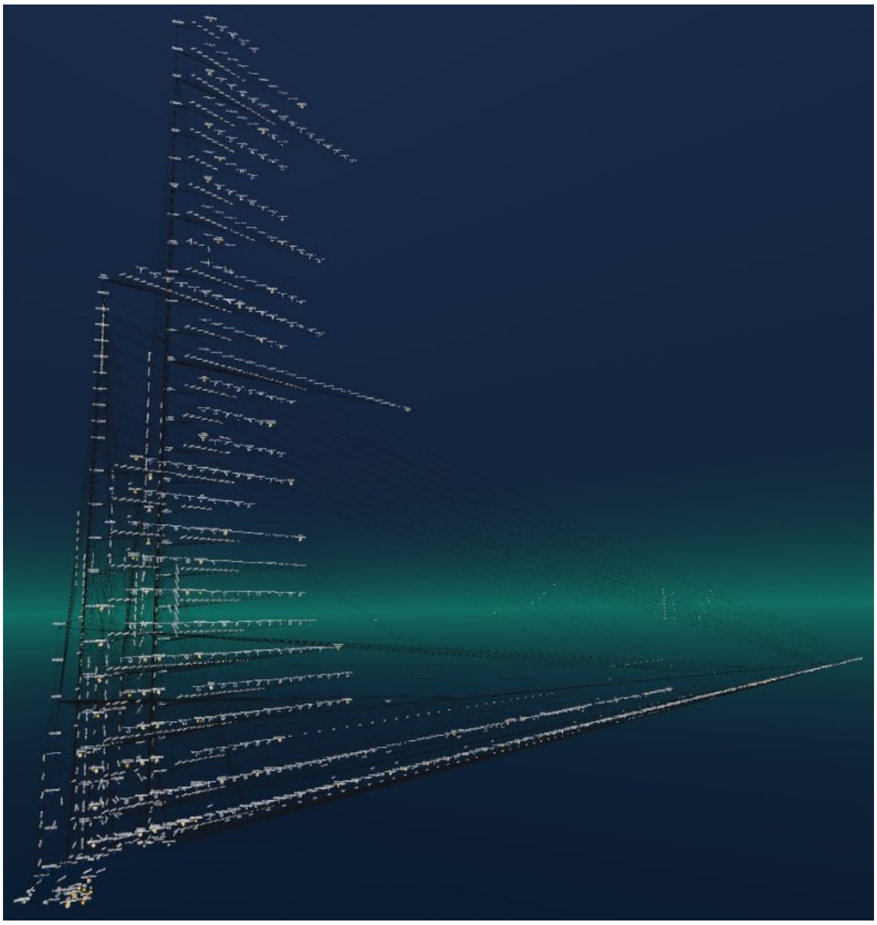

Figure C.13: Graph2VR layout of the complete dataset with additional triples about the class hierarchy. The layout is mostly generated automatically, with only a few nodes that clustered at the same place in the lower left corner needing to be adjusted manually.

The class hierarchy layout needs the relationships between classes and subclasses (rdfs:subclassOf), as well as the relation between class and individuals (rdfs:type), to be defined as such. Additionally, it is helpful to add the rdfs:subclassOf predicate to connect classes that do not have another superclass to owl:thing. For example, for patients, this can be archived by adding these triples:

```
sio:SIO_000393 rdf:type owl:Class ;
               rdfs:subClassOf owl:Thing ;
               rdfs:label "patient" .
```

Appendix D. Supplementary information on use case 3

Appendix D.1. Relevant URIs for use case 3

Especially for PubChem, due to its size, it is relevant to know which graphs are necessary to load. To determine which graphs are relevant, it is possible to refer to the database schema in the paper ”PubChemRDF: towards the semantic annotation of PubChem compound and substance databases” [39]. It describes the relations of the different graphs of PubChem. Despite its size, the schema behind those graphs is relatively small. The structure of the graphs has barely changed since the paper was published, only some URIs from external ontologies, especially the sio ontologies, have been updated. As an example, here are the predicated used to describe entities of the compounds graph:

**URI**	**Description**
http://www.w3.org/1999/02/22-rdf-syntax-ns#type	type
http://purl.org/dc/terms/modified	Date Modified
http://purl.org/spar/cito/isDiscussedBy	The cited entity presents statements, ideas or conclusions that are discussed by the citing entity.
http://semanticscience.org/resource/SIO_000008	has attribute
http://www.w3.org/2004/02/skos/core#exactMatch	has exact match
http://purl.org/dc/terms/source	Source
http://purl.obolibrary.org/obo/RO_0000056	participates in
http://rdf.wwpdb.org/schema/pdbx-v40.owl#link_to_pdb	link_to_pdb
http://semanticscience.org/resource/CHEMINF_000477	Has PubChem normalized counterpart. Non-symmetric predicate between substance as domain b and compound as range c
http://purl.org/dc/terms/available	Date that the resource became or will become available. - Recommended practice is to describe the date, date/time, or period of time as recommended for the property Date, of which this is a subproperty.

Table D.2: Description of RDF predicates in PubChem

For use case 3, we connected Dutch drug names to Drugbank and PubChem, so we had to combine URIs from all 3 databases. The relevant graphs from PubChem were substances, compounds, and synonyms since they contain data about chemical substances. The relevant URIs used in use case 3 are:

**URI**	**Description**
http://www.w3.org/2000/01/rdf-schema#label	”label”
http://www.w3.org/2002/07/owl#sameAs	”sameAs”
http://www.w3.org/1999/02/22-rdf-syntax-ns#type	”type”
http://www.w3.org/2002/07/owl#Class	”Class”
http://bio2rdf.org/bio2rdf_vocabulary:identifier	”Bio2RDF_identifier”
http://bio2rdf.org/drugbank_vocabulary:x-atc	”drugbank_vocabulary:x_atc” (ATC code)
http://bio2rdf.org/drugbank:DB00945	”Acetylsalicylic acid [drugbank:DB00945]”
http://bio2rdf.org/atc:B01AC06	”B01AC06” (ATC code of Aspirin)
http://semanticscience.org/resource/SIO_000011	”is attribute of”
http://semanticscience.org/resource/SIO_000300	”has value of”
http://semanticscience.org/resource/CHEMINF_000406	”Drugbank identifier”
http://semanticscience.org/resource/CHEMINF_000446	”CASNO” (Chemical Abstracts Service number)

Table D.3: Description of RDF predicates in Bio2RDF and Drugbank

Appendix E. Supplementary overview Graph2VR Settings file options

Table E.4: Overview of Graph2VR Settings File Options
Graph2VR allows users to customize their experience through a configurable settings file, named settings.txt. This JSON-formatted file provides various setting options, which are detailed below. A template of such a settings file can be found on the Graph2VR GitHub repository [8].

Option	Description and Example Value
`sparqlEndpoint`	Defines which SPARQL Endpoint should be used when sending SPARQL queries. **Example:** `"https://dbpedia.org/sparql"`
`baseURI`	The graph can optionally be specified if the entry is empty, then all graphs are selected, at least, this is the default behaviour when using a Virtuoso server. **Example:** `""`
`databaseSupportsbif:contains`	This option specifies whether Graph2VR should use the bif:contains command in search queries for this SPARQL Endpoint. The option bif:contains is only supported by some servers. It improves search speed by using some queries over the internal SQL database. However, having a server that supports the command is not enough. The server must also have built an index of its data to be able to support the command. If no index is built, the result will most likely be an empty result. DBpedia has built such an index, so the faster search method can be used. **Example:** `true`
`defaultNodeCreationURI`	These define the URIs of newly created nodes, and a counter is added to it. **Example:** `"http://graph2vr.org/newNode#"`
`defaultEdgeCreationURI`	Defines the URIs for newly created edges and a counter is added to it. **Example:** `"http://graph2vr.org/newEdge#"`

<table>
<tr>
<td><code>initialSparqlQueryString</code></td>
<td>

This option defines the initial SPARQL query and, therefore, how the initial Graph in Graph2VR looks. It is important to know that this query must be a CONSTRUCT query; SELECT queries do not work properly. Given that 'settings.txt' is formatted as a JSON file, special characters must be correctly escaped. Tools like a JSON Encoder simplify this task by ensuring proper character encoding [17]. This starting query may contain options like Minus, Bind, subqueries etc., even if Graph2VR's GUI does not yet have them. This SPARQL query will be executed by DotNetRDF, and the resulting graph will be displayed.
Example:

```
"prefix dbo: <http://dbpedia.org/ontology/>
prefix rdfs: <http://www.w3.org/2000/01/rdf-schema#>
prefix rdf: <http://www.w3.org/1999/02/22-rdf-syntax-ns#>
prefix dbp: <http://dbpedia.org/property/>
prefix dbr: <http://dbpedia.org/resource/>
construct {
  ?mountain rdf:type dbo:Mountain .
  ?mountain dbo:elevation ?height .
  ?mountain dbp:location ?location .
  ?mountain rdfs:label ?label .
  ?mountain <http://xmlns.com/foaf/0.1/depiction> ?image .
}
where {
  ?mountain rdf:type dbo:Mountain .
  ?mountain dbo:elevation ?height .
  ?mountain dbp:location ?location .
  ?mountain rdfs:label ?label .
  ?mountain <http://xmlns.com/foaf/0.1/depiction> ?image .
}
ORDER BY DESC(?height) LIMIT 50"
```

</td>
</tr>
<tr>
<td><code>initialSparqlURI</code></td>
<td>

Alternatively, it is possible to start with a single URI as starting Graph.
Example:

```
"http://www.w3.org/2002/07/owl\#Thing"
```

</td>
</tr>
<tr>
<td><code>startWithSingleNode</code></td>
<td>When the option ”startWithSingleNode” is set as false, the query will be used to generate the starting graph. If it is set to true, the ”initialSparqlURI” will be used to generate a graph consisting only of this single node instead.
Example: <code>false</code></td>
</tr>
<tr>
<td><code>searchOnKeypress</code></td>
<td>If ”searchOnKeypress” is true, the search will be triggered when typing on the keyboard (for more than three letters). If it is set to false, the return key needs to be pressed on the virtual keyboard to trigger the search.
Example: <code>true</code></td>
</tr>
<tr>
<td><code>playerHeight</code></td>
<td>This is a parameter to configure the height of the headset above the ground at the beginning.
Example: <code>1.7999999523162842</code></td>
</tr>
</table>

`imagePredicates`	Depending on the image predicates, Graph2VR determines what predicates indicate that an image is expected and tries to fetch it.<br>**Example:**<br>`["http://xmlns.com/foaf/0.1/depiction",`<br>`"http://xmlns.com/foaf/0.1/Image",`<br>`"http://xmlns.com/foaf/0.1/thumbnail",`<br>`"http://dbpedia.org/property/photo",`<br>`"http://dbpedia.org/ontology/thumbnail"`<br>`]`
`infopanelPredicates`	Since some nodes have many entries, we decided not to load all of them in the info panel. Instead, only some preselected predicates are displayed.<br>**Example:**<br>`[`<br>`"http://www.w3.org/1999/02/22-rdf-syntax-ns\#type",`<br>`"http://www.w3.org/2000/01/rdf-schema\#label"`<br>`]`
`literalColor`	Specifies colors for various elements in Graph2VR. VOWL schema was the blueprint for the colouring schema of the nodes. Even though there are templates, not all of them have actually been implemented. Literals, for example, are yellow.<br>**Example:**<br>`"literalColor": {`<br>`"r": 1.0,`<br>`"g": 0.800000011920929,`<br>`"b": 0.20000000298023225,`<br>`"a": 1.0`<br>`}`
`nodeOwlClassColor`	OwlClasses are light blue. (the colour is only shown when the relationship is present in the graph)<br>**Example:**<br>`"nodeOwlClassColor": {`<br>`"r": 0.6666666865348816,`<br>`"g": 0.800000011920929,`<br>`"b": 1.0,`<br>`"a": 0.0`<br>`}`

`nodeRdfsClassColor`	Rdfs classes are purple<br>**Example:**<br><br>`"nodeRdfsClassColor": {`<br>`    "r": 0.800000011920929,`<br>`    "g": 0.6000000238418579,`<br>`    "b": 0.800000011920929,`<br>`    "a": 0.0`<br>`}`
`nodeOwlDatatypeColor`	Owl datatypes are green<br>**Example:**<br><br>`"nodeOwlDatatypeColor": {`<br>`    "r": 0.6000000238418579,`<br>`    "g": 0.800000011920929,`<br>`    "b": 0.4000000059604645,`<br>`    "a": 0.0`<br>`}`
`deprecatedColor`	Deprecated nodes are grey<br>**Example:**<br><br>`"deprecatedColor": {`<br>`    "r": 0.8,`<br>`    "g": 0.8,`<br>`    "b": 0.8,`<br>`    "a": 1.0`<br>`}`
`arrowheadSubclassOfColor`	Usually arrowheads have the same colour as the arrow itself, but if it is a subclass of relation, the arrowhead is white.<br>**Example:**<br><br>`"arrowheadSubclassOfColor": {`<br>`    "r": 1.0,`<br>`    "g": 1.0,`<br>`    "b": 1.0,`<br>`    "a": 1.0`<br>`}`

`databaseSetttings`	When working with Graph2VR, it might be desirable to not only work with the initial graph database but to also switch between multiple databases. This allows the extension of the currently active graph with nodes and edges from a different database. In the case of matching URIs, this can be used for expansion as well.<br>`{`<br>`"label": "Disgenet",`<br>`"sparqlEndpoint": "http://rdf.disgenet.org/sparql/",`<br>`"baseURI": "http://rdf.disgenet.org/",`<br>`"databaseSupportsbif:contains": false,`<br>`"searchOnKeypress": false`<br>`},`
`predefinedPredicates`	Allows the predefined predicates to be used from the menu in VR, enhancing usability. The selection of predicates is based on a suggestion of predicates for comparison based on a paper about ”Scientific Lenses”.<br>**Example:**<br>`"predefinedPredicates": [`<br>`{`<br>`"name": "owl:sameAs",`<br>`"uri": "http://www.w3.org/2002/07/owl#sameAs"`<br>`},`<br>`{`<br>`"name": "skos:exactMatch",`<br>`"URI":`<br>`"http://www.w3.org/2004/02/skos/core#exactMatch"`<br>`},`<br>`{`<br>`"name": "skos:closeMatch",`<br>`"URI":`<br>`"http://www.w3.org/2004/02/skos/core#closeMatch"`<br>`},`<br>`{`<br>`"name": "rdfs:seeAlso",`<br>`"URI":`<br>`"http://www.w3.org/2000/01/rdf-schema#seeAlso"`<br>`}`<br>`],`
`savedMaximumImageWidth`,<br>`savedMaximumImageHeight`	Defines the maximum dimensions for images stored in .g2v files to save memory. Larger images are scaled down.<br>**Example:** `256, 256`
